# COVID-19 and Student Well-Being: Stress and Mental Health during Return-to-School

**DOI:** 10.1177/08295735211001653

**Published:** 2021-03-18

**Authors:** Kelly Dean Schwartz, Deinera Exner-Cortens, Carly A. McMorris, Erica Makarenko, Paul Arnold, Marisa Van Bavel, Sarah Williams, Rachel Canfield

**Affiliations:** 1 University of Calgary, AB, Canada

**Keywords:** COVID-19, secondary education/adolescence, social and educational environment, health and wellbeing, coping, mental health, stress, high school, participants, junior high school

## Abstract

Students have been multiply impacted by the COVID-19 pandemic: threats to their own and their family’s health, the closure of schools, and pivoting to online learning in March 2020, a long summer of physical distancing, and then the challenge of returning to school in fall 2020. As damaging as the physical health effects of a global pandemic are, much has been speculated about the “second wave” of mental health crises, particularly for school-aged children and adolescents. Yet, few studies have asked students about their experiences during the pandemic. The present study engaged with over two thousand (*N* = 2,310; 1,288 female; *M*_age_ = 14.5) 12- to 18-year-old Alberta students during their first few weeks of return-to-school in fall 2020. Students completed an online survey that asked about their perceptions of COVID-19, their fall return-to-school experiences (84.9% returned in-person), their self-reported pandemic-related stress, and their behavior, affect, and cognitive functioning in the first few weeks of September. The majority of students (84.9%) returned to school in person. Students reported moderate and equal concern for their health, family confinement, and maintaining social contact. Student stress levels were also above critical thresholds for 25% of the sample, and females and older adolescents (age 15–18 years) generally reported higher stress indicators as compared to males and younger (age 12–14 years) adolescents. Multivariate analysis showed that stress indicators were positively and significantly correlated with self-reported behavioral concerns (i.e., conduct problems, negative affect, and cognitive/inattention), and that stress arousal (e.g., sleep problems, hypervigilance) accounted for significant variance in behavioral concerns. Results are discussed in the context of how schools can provide both universal responses to students during COVID-19 knowing that most students are coping well, while some may require more targeted strategies to address stress arousal and heightened negative affect.

The words “unprecedented,” “historic,” and “crisis” are tired words that have exhausted their usefulness in describing the COVID-19 worldwide pandemic. For students who had their school years halted in mid-March 2020, however, these words likely hold true as their access to classroom instruction, peer groups, teacher-mentors, and academic support were abruptly made unavailable, and for many youth remain disrupted. For students who returned to school in the fall of 2020, classrooms and school buildings looked and felt very different: physical distancing was required among students and teachers, personal protective equipment (e.g., masks) was often mandated; students may have experienced modified and changing curriculum delivery; and students may have developed a heightened awareness of their own physical health. Given these disruptions, much has been speculated about the impact of the pandemic on academic achievement, peer and friend relationships, and mental health and well-being among children and youth (Racine, Korczak et al., 2020). With respect to the latter, this includes a fear that the ”second wave” of the pandemic will be in the form of dramatic increases in mental health problems.

As the world enters various phases of recovery from COVID-19, millions of North American students entered and exited their summers unsure what to expect when schools re-opened, in innumerable forms and employing diverse delivery platforms, in fall 2020. As there is much interest in the mental and behavioral health of students as they re-engage with their schools, curricula, teachers, and peers, the present study explored two general questions related to COVID-19 school re-entry in the Canadian context: (1) What are the lived experiences of secondary students related to COVID-19, including their concerns about personal, family, and national health, and their schooling experience at present and during lockdown?; and (2) How is student COVID-19-related stress correlated with and predictive of self-reported mental health indicators (e.g., conduct, negative affect, cognitive/attention)?

## Global Events and Adolescent Mental Health

Although the existing literature is limited, how adolescents have experienced and are currently being impacted by other national and international natural disasters and events is important to consider as we strive to understand the potential impacts of COVID-19 on youth mental health and well-being. Past work in this area tends to explore stable areas of developmental psychology inquiry, namely the age and gender differences that are common in response to significant stressors (e.g., Dunn et al., 2017). Prior to the global pandemic, only a small body of research looked at stress responses in relation to health-related disasters, and much of this research has focused on how young people react to trauma associated with natural disasters such as hurricanes, tornados, floods, and fires. Although these disasters differ from a pandemic in many ways (e.g., degree of separation and isolation, required quarantine), given that these disasters also involve widespread community impact, fatalities, and unpredictability, they are still applicable in the COVID-19 context (Sprang & Silman, 2013).

Studies exploring adolescents’ responses to past disasters have generally found poorer mental health for some sub-groups following the event. For example, studies exploring the aftermath of Hurricane Katrina found that adolescent females reported significantly higher symptoms of depression and posttraumatic stress disorder (PTSD), and that they also reported higher rates of re-experiencing and avoidance than males (Kronenberg et al., 2010). Gender differences seem to remain over time, as female adolescents have also been found to show higher levels of distress 28 months after experiencing a disaster (Bokszczanin, 2007). However, such findings are not consistent across the literature. For example, Adams et al. (2014) did not find a difference in the number of males and females who met criteria for a diagnosis of PTSD following a tornado outbreak in major regions of the United States. Although few studies specifically explore traumatic stress responses of children to prior pandemics, Sprang and Silman (2013) examined adolescent responses to the 2009 H1N1 pandemic. Using parent-reported symptoms of posttraumatic stress, they found that over one-third of children who experienced isolation or quarantine demonstrated symptoms that met the overall diagnostic threshold for PTSD; however, no gender differences were found.

## Adolescents and COVID-19

Now almost 12 months into the COVID-19 pandemic, the literature on the mental health of adolescents during the pandemic is growing rapidly (Racine, Cooke et al., 2020). Recent reports and surveys completed during the pandemic have provided concerning information regarding how students are coping with and adapting to school closures, physical distancing, and quarantining time at home. Some have reported that food insecurity, gaps in math and literacy skills, unreliable internet access, and precarious housing situations are the new reality (Van Lancker & Parolin, 2020), while others anticipate that children and youth may experience increased stress and anxiety related to the COVID-19 pandemic (Orgiles et al., 2021; Xie et al., 2020). However, closer to home, a recent national survey of Canadian youth (age 10–17 years) found that a significant proportion of youth had typical responses regarding their views, experiences, and opinions of COVID-19. For example, many adolescents reported being generally bored (71%), feeling quite normal (41%), missing their friends (54%), being academically unmotivated (60%), and generally were disliking their current social isolation (57%; Korzinski, 2020). Thus, far from showing evidence of a pending crisis in mental health, early Canadian results, in general, represent a reaction of adolescents to major shifts in their social, familial, and school arenas that are both expected and developmentally appropriate.

Canadian research on COVID-19 and its impact on mental health and other outcomes is only now starting to be released. For example, Hawke et al. (2020) examined mental health among youth with and without physical health challenges during the early stages of COVID-19. Participants in their sample of 14 to 28 year-olds indicated that youth with pre-existing physical health conditions (e.g., asthma, diabetes) and those with symptoms associated with COVID-19 (i.e., fever, shortness of breath, cough/sore throat) reported more impact on their mental health and physical health than those without pre-existing mental and/or physical health concerns. Of note, those respondents who reported symptoms more commonly associated with a common cold also reported higher mental health symptoms (e.g., behavioral and attention concerns, anxiety and depression symptoms, substance use). Craig et al. (2020) also explored current rates of mental health problems and COVID-19 related stress in Canadian youth; they found that females endorsed significantly higher levels of depression, anxiety, social phobia, and PTSD than males. As the data were collected during the early stages of quarantine and school closures (June 2020), the findings may represent the initial effects of social isolation and family confinement on adolescent mental health, as much as concerns over the health effects of COVID-19 itself. Indeed, Ellis et al. (2020) found that, in their sample of 14- to 18-year-olds, stress-related to COVID-19 was associated with more loneliness and higher symptoms of mental health disorders (e.g., depression), while those who spent more time with family and were more focused on schoolwork had fewer self-reported mental health symptoms. Thus, important questions remain about the impacts of COVID-19 stress itself, as compared to related factors (school closure, family confinement), in understanding mental health outcomes for youth in Canada.

## School Attendance and Student Mental Health

Many schools across North America physically closed in March 2020 as a precautionary measure in response to the rapid spread of COVID-19. This sudden shift from physical school attendance and regular interaction with peers and teachers to online learning and quarantining at home was a difficult adjustment for many learners across grade levels (Magson et al., 2020). Decades of research have provided support for the importance of physical school attendance on adolescent mental health. For example, absenteeism and poor mental health have a bidirectional relationship, with poorer reported mental health in adolescents leading to increased absenteeism (Lawrence et al., 2019), and chronic absenteeism resulting in decreased physical and mental health outcomes for children and adolescents (Wood et al., 2012). The importance of attending school in person is also highlighted by the fact that many children and youth also receive mental health services while they are physically present at school (Duong et al., 2021).

Adolescence has been recognized as a critical time for the development of social relationships and the need for peer interaction, and it is during this period that adolescents shift from primarily spending time with parents to an increased influence and time spent with peers (Meuwese et al., 2017; Steinberg, 2020). In addition, school attendance and school connectedness have been identified as protective factors for children and youth against a range of poor physical and mental health outcomes (Bond et al., 2007). Because school is the place where adolescents spend a significant amount of time with peers, the shift to online learning in March 2020 may have been more difficult and possibly detrimental to adolescent mental health and resilience in particular due to these factors.

## Purpose of the Present Study

To determine how COVID-19 has impacted adolescents’ mental health and well-being, the goal of the present study was to gather responses from students regarding their feelings about COVID-19 and related health and protective behaviors, their stress related to COVID-19, and their self-reported mental health. Based on prior disaster-related literature and emerging COVID-19 findings, it was expected that the majority of students would report making adequate adjustments to school closures, report mild to moderate impacts of stress related to COVID-19, and report moderate but clinically insignificant levels of social, emotional, and behavioral functioning. Consistent with previous research, we anticipated that females and older youth would report higher stress and higher symptoms of mental health problems, and that those with higher self-reported stress would also report higher levels of poor mental health. Controlling for age and gender, COVID-related stress was expected to account for significant and negative mental and behavioral health outcomes.

## Method

### Participants

A total of 5,277 parents from four Alberta metropolitan school divisions (two public, two Catholic) provided consent for their child (ages 12–17 years) to participate in an online survey; an additional 109 students who were age 18 years or older provided their own consent to participate in the survey. In total, 2,425 students completed all or some of the online survey (46% response rate); 115 students did not complete enough items for analysis in the present study, resulting in a final sample of 2,310 participants.

### Measures

#### Remote learning experience

Students were asked to respond to questions related to their education following March 2020 school closures. This included asking about which support services they had received (e.g., family counseling, individual counseling, group counseling), engagement with their teacher(s) (e.g., online, in-person/online, in-person), and parent engagement with remote learning since school closure (e.g., setting up a daily schedule, asking about schoolwork, etc.). Finally, students were asked in what way they returned to school in fall 2020 (i.e., in-person, online/virtual, both in-person, and online/virtual).

#### COVID-19 health and protection behaviors

COVID-19 questions were related to physical (2-m) distancing, socializing outside one’s bubble, mask-wearing, decisions regarding wearing of personal protective equipment (PPE; i.e., masks), and exposure to media related to COVID-19. Questions were also asked about participants’ concerns (from 1-*not at all* to 4-*extremely*) related to their own health, household members’ health, vulnerable peoples’ health, nation and world health, overloading the health system, maintaining social ties, family confinement and stress, and violence in the home.

#### Child Revised Impact of Events Scale (CRIES)

The CRIES is a 13-item measure designed to assess present experiences of a traumatic event, avoidance of that event, and the feelings to which it gave rise (Weiss & Mamar, 1997). Participants were asked to respond to items by rating “how frequently these comments were true for you during the past 7 days about COVID-19” (0 = *Not at all*, 1 = *Rarely*, 3 = *Sometimes*, and 5 = *Often*). The CRIES produces a Total score (possible score range 0–65; α = .90) and three subscales: Intrusion (four items, e.g., “Do you think about COVID-19 even when you don’t mean to?”; possible score range 0–20; α = .80), Avoidance (four items, e.g., “Do you try not to think COVID-19?; possible score range 0–20; α = .82), and Arousal (five items, e.g., “Do you have sleep problems?”; possible score range 0–25; α = .78). For the Total scale and all subscales, higher scores indicate more distress. In prior research with youth, a Total score above 30 and subscale scores above 17 have been found to identify increased risk of posttraumatic stress (e.g., Giannopoulou et al., 2006). In the present study, the CRIES is used as an estimate of self-reported posttraumatic stress reactions (e.g., Total Score and Intrusion, Avoidance, Arousal subscales) with specific reference to COVID-19.

#### Behavior Intervention Monitoring Assessment System (BIMAS-2)

The BIMAS-2 is a 34-item universal screening measure of conduct problems, negative affect, and reduced cognitive/attention functioning (McDougal et al., 2011). Participants were asked: “Please rate how often each of the following behaviors occurred during the past week,” with response options provided from 0 = *Never (0 times)* to *4* = *Very often (Occurred 7 or more times or to an extreme extent)*. The BIMAS self-report raw scores were then converted to standardized *T*-scores, which have a mean of 50 and standard deviation of 10. Three Behavioral Concern Scales are produced: Conduct (nine items; e.g., “During the past week, I . . . felt angry”; α = .74), Negative Affect (seven items; e.g., “During the past week, I . . . was sad or withdrawn”; α = .89), and Cognitive/Attention (seven items; e.g., “During the past week, I . . . had trouble remembering things”; α = .86). Within the three Behavioral Concern Scales, higher scores indicated more concerns; a *T*-score of 70+ is described as High Risk, *T*-scores between 60 and 69 are described as Some Risk, and *T*-scores under 60 are described as Low Risk (McDougal et al., 2011).

#### Demographics

Included were age; gender; race/ethnicity (White, Black, Asian, Southeast Asian, Latin, Indigenous, Other, I prefer not to answer); family structure; and socioeconomic status (based on highest level of parental education, eighth grade or less to post-university study). For race/ethnicity, based on write-in responses to the “Other” category, an Arab/Middle Eastern group was also created.

### Recruitment Procedure

Ethics approval was received from a university research ethics board. The four school divisions also approved the study. Study announcements were provided to all school divisions and distributed to parents/guardians and students aged 18 years and older via directs e-mails during the first week of return-to-school (approximately September 2–5, 2020). Once they had read about the purpose of the study, parents/guardians were asked to electronically indicate their permission for their child(ren) to participate in the study; if they responded affirmatively, they were subsequently asked to provide a student e-mail address to which the survey link would be sent. Home (56%) and cell phone (65.7%) numbers were also provided by the parent for follow-up contacts for subsequent waves of data collection. Student participants who received the survey e-mail were provided information about the study and were also asked for their expressed assent to participate in the study. Following the initial email, students were sent up to two reminder emails with the survey link. The survey was closed on October 2, 2020. Students who completed the survey received a $10 gift card as a thank you for participating. All surveys were completed via REDCap.

## Results

### Description of the Sample

The mean (SD) age of the 2,310 participants in this study was 14.56 (1.78). Just over half of participants (55.8%) identified as female, with the remainder identifying as male (41.0%) and non-binary/trans/other (1.3%). There was not a significant difference in gender by age. The majority of participants identified as White only (63.0%), followed by Asian only (11.9%), multi-ethnic (7.8%), Southeast Asian only (4.1%), Black only (3.3%), Latin only (2.5%), Arab/Middle Eastern only (1.5%), Indigenous only (1%), and Other (2.1%); 2.6% of participants preferred not to say. Family structure was reported as follows: living in a two-parent household (all family members biologically related, 77.2%), single-parent mother household (7.8%), split time between two parents (7.1%), two-parent household step-family (3.2%), two-parent blended family (1.4%), and other (2.0%). Finally, the mean (SD) socioeconomic status in our sample was 4.97 (0.84), or an average parental level of education between some post-secondary and university graduate.

### Remote Learning Experiences

In fall 2020, four out of five participants (80.4%) indicated that, when schools closed in March 2020, they engaged with their teachers and/or the school curriculum in an online/remote learning environment; 14% connected both in-person and online, 1.9% in-person only, and 3.6% did not engage at all with their teachers. When asked how their parents, guardians, and/or caregivers engaged with this remote learning in March, almost two out of three students (64.9%) indicated that their caregivers asked about their schoolwork, while just under half (46.4%) reported that they had mostly directed their own learning. Considering support services received by the participants, most students (85.1%) indicated that they received no external therapeutic support after schools closed; however, approximately 1 in 10 (10.3%) did seek individual counseling or therapy in the months following school closures. Finally, when schools reopened in September 2020, the majority of participants (84.0%) reported that they returned to school in-person, while 11.9% remained in an online learning setting. See Table 1 for descriptive results on these learning experiences.

**Table 1.** Remote Learning Experiences (*N* = 2,310).

Question	% (*n*)
Teacher/school engagement since March	
Online	80.4 (1,858)
Hybrid	14.0 (323)
None	3.6 (83)
In-person	1.9 (43)
Parent/guardian/caregiver learning engagement since March^a^	
Asking about schoolwork	64.9 (1,500)
I have mostly directed own learning	46.4 (1,077)
Helping me with schoolwork	39.1 (903)
Setting up a daily schedule	28.5 (659)
Limiting non-school activities	17.3 (399)
Support services received since March^a^	
None	85.1 (1,965)
Individual	10.3 (238)
Family	5.0 (115)
Group	1.4 (33)
In what way did you return to school in fall 2020?	
In-person	84.0 (1,941)
Online	11.9 (275)
Hybrid	3.9 (89)
Did not return	0.2 (5)

a Participants could select more than one response option.

### COVID-19 Health and Protective Behaviors

In response to questions related to physical and social distancing, participants were uniformly quite careful about ensuring their distance in the past month. Overall, 90.7% of participants reported wearing a mask in public all or most of the time (though we note that both areas where participants were recruited from had mask mandates in place), and 64.8% of participants stated that they engaged in physical distancing all or most of the time (Table 2). Looking at differences by gender, males were significantly more likely to say that they maintained a 2-m distance with people outside of their household most of the time, while females were more likely to state that they did this rarely (χ^2^(4, *N* = 2,228) = 14.82, *p* = .005). However, females were more likely than males to say that they wore a mask in public all the time χ^2^(4, *N* = 2,232) = 12.91, *p* = .012). With respect to age, significant differences were also found in physical distancing, with younger adolescents more likely to state they complied all or most of the time with statements about physical distancing (*F*(4, *N* = 2,303) = 8.63, *p* < .001) and maintaining a 2-m distance with people outside of their household (*F*(4, *N* = 2,301) = 5.43, *p* < .001), as compared to older adolescents. Conversely, 15- to 18-year-olds endorsed socializing with someone outside their bubble significantly more than 12 to 14 year-olds (*F*(1, 2,306) = 8.49, *p* < .01). See Table 2 for scores by gender (male, female) and age.

**Table 2.** COVID-Related Health Questions (*N* = 2,310).

	Overall	Gender^a^			Age	
	% (*n*)	Male, % (*n*)	Female, % (*n*)	*p* Value	Mean (*SD*)	*p* Value
In the past month, to what extent did you engage in physical distancing?						
All of the time	13.2 (306)	14.1 (133)	12.6 (162)	.633	14.43 (1.85)	<.001
Most of time	51.6 (1,191)	52.4 (496)	51.1 (656)		14.44 (1.75)	
Some of the time	23.5 (544)	22.4 (212)	24.4 (313)		14.64 (1.79)	
Not most of the time	9.3 (215)	8.9 (84)	9.9 (127)		15.00 (1.64)	
Not at all	2.2 (50)	2.2 (21)	2.1 (27)		15.44 (1.79)	
In the past month, how often did you socialize in person with someone outside your immediate household or allowable social bubble?						
A great deal	19.0 (438)	20.0 (189)	18.4 (237)	.126	14.71 (1.68)	.078
A lot	17.1 (394)	17.1 (162)	17.3 (223)		14.68 (1.80)	
Somewhat	29.7 (686)	28.1 (266)	30.9 (398)		14.49 (1.80)	
A little	27.1 (626)	26.2 (248)	27.4 (353)		14.50 (1.80)	
Not at all	7.1 (165)	8.5 (80)	6.0 (77)		14.38 (1.78)	
In the past month, when you saw people outside of your household, how often did you maintain 2-m distance?						
All of the time	15.4 (355)	16.6 (157)	14.5 (186)	.005	14.41 (1.79)	<.001
Most of the time	43.5 (1,006)	46.7 (440)	40.9 (526)		14.43 (1.77)	
Some of the time	25.3 (585)	23.2 (219)	27.1 (348)		14.72 (1.78)	
Rarely	12.0 (278)	10.3 (87)	13.8 (177)		14.84 (1.68)	
Not at all	3.5 (80)	3.2 (30)	3.7 (48)		14.80 (1.89)	
In the past month, to what extent did you wear a mask in public?						
All of the time	60.1 (1389)	56.5 (533)	62.6 (805)	.012	14.60 (1.77)	.235
Most of the time	30.6 (706)	32.9 (311)	29.2 (376)		14.44 (1.75)	
Some of the time	6.8 (158)	7.9 (75)	6.1 (79)		14.68 (1.89)	
Rarely	2.1 (48)	2.3 (22)	1.9 (25)		14.79 (1.75)	
Not at all	0.3 (6)	0.5 (5)	0.1 (1)		15.00 (2.53)	

a Number of trans/non-binary/other participants too small to include in analyses.

### Stress and Mental Health

#### Descriptive and bivariate analysis

Overall, using the CRIES, participant stress reactions were found to be well below the critical threshold of 30 (*M* = 20.00, *SD* = 14.58). In addition, each of the mean subscale scores was well below the critical threshold of 17: Intrusion (*M* = 5.85, *SD* = 5.09), Avoidance (*M* = 5.98, *SD* = 5.69), and Arousal (*M* = 8.20, *SD* = 6.27). See Table 3 for descriptive results for the Total and subscale scores by gender and age. Without exception, female participants had significantly higher Total and subscale scores than male participants, and older youth (age 15–18 years) rated all Total and subscale scores significantly higher than did younger youth (age 12–14 years).

**Table 3.** CRIES Subscale and BIMAS Behavior Concern Scale Scores, Overall and by Gender and Age (*N* = 2,310).

	Overall, mean (*SD*)	Gender^a^			Age		
		Male, mean (*SD*)	Female, mean (*SD*)	*p* Value	12–14 years, mean (SD)	15–18 years, mean (*SD*)	*p* Value
CRIES subscales							
Total score	20.0 (14.69)	15.89 (13.29)	22.79 (14.87)	<.001	17.50 (13.89)	22.54 (15.11)	<.001
Intrusion subscale	5.85 (5.09)	4.64 (4.72)	6.68 (5.17)	<.001	5.05 (4.80)	6.67 (5.26)	<.001
Avoidance subscale	5.98 (5.69)	4.89 (5.42)	6.72 (5.75)	<.001	5.62 (5.65)	6.36 (5.74)	<.001
Arousal subscale	8.20 (6.27)	6.98 (5.45)	9.39 (6.45)	<.001	6.84 (5.81)	9.52 (6.45)	<.001
BIMAS behavior concern scales							
Conduct	48.93 (6.92)	48.13 (7.07)	49.46 (6.68)	<.001	49.23 (6.48)	48.64 (7.31)	.042
Negative affect	58.40 (11.33)	53.80 (10.63)	61.60 (10.57)	<.001	56.89 (11.02)	59.87 (11.42)	<.001
Cognitive/attention	55.32 (10.18)	53.36 (10.16)	56.55 (9.83)	<.001	54.65 (10.31)	55.98 (10.01)	.002

a Number of trans/non-binary/other-gender participants too small to include in analyses.

**Figure 1.**
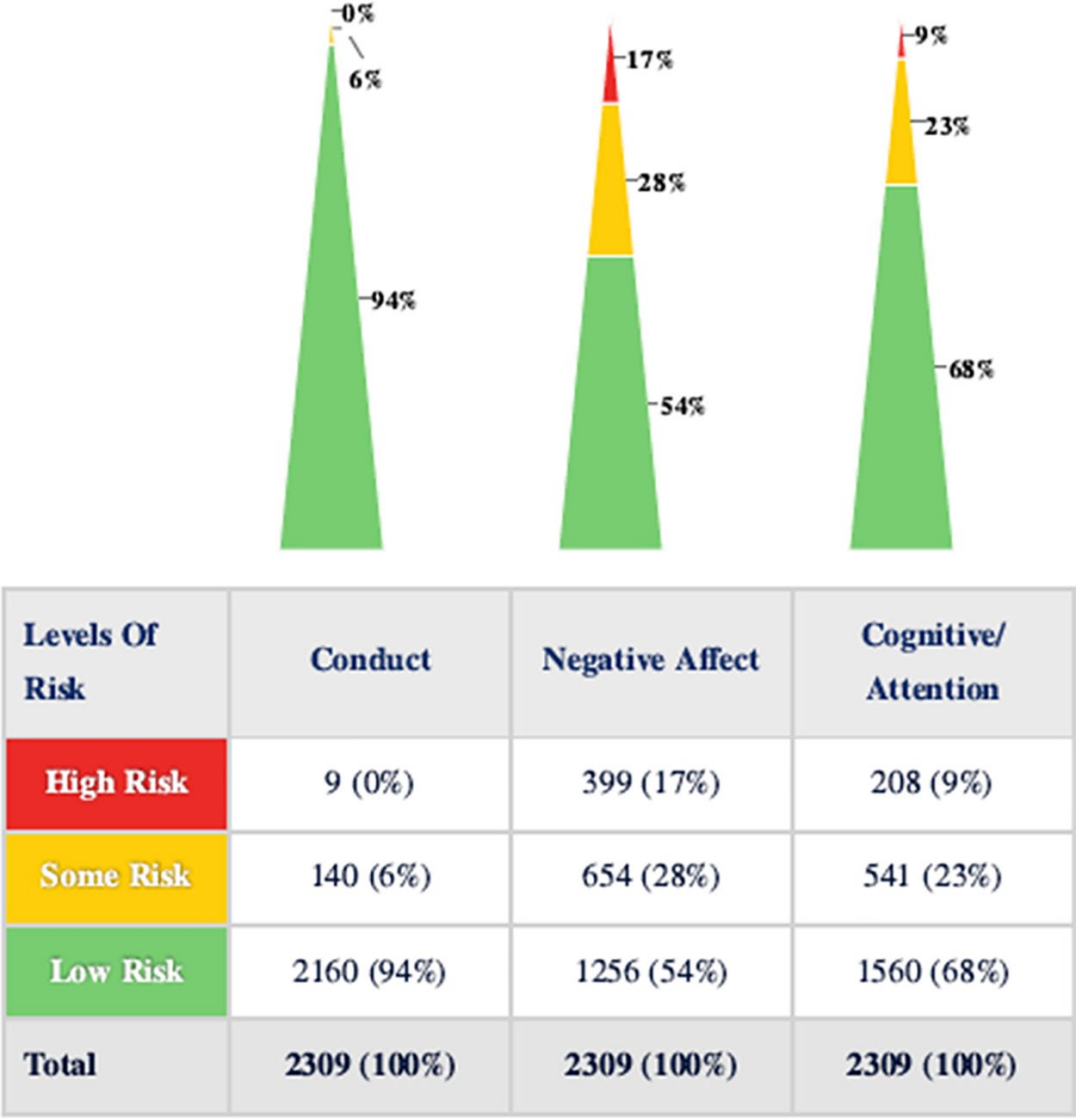
BIMAS behavioral concern risk levels.

On the BIMAS, Behavioral Concern Scale (BCS) *T*-scores were also found to be in the low risk range overall, with mean (SD) scores of 48.93 (6.92) for Conduct, 58.40 (11.33) for Negative Affect, and 55.32 (10.18) for Cognitive/Attention scales. See Table 3 for descriptive results of BCS scores by gender and age. However, 17% of students’ *T*-scores were in the high risk range for the Negative Affect scale, and 9% of students’ ratings on the Cognitive/Attention scale fell in the high risk range. No students’ *T*-scores were in the high risk range on the Conduct scale (Figure 1).

### Stress and Mental Health

Examining partial correlations (controlling for age and gender) between the three Behavioral Concern Scales (BCS) and the CRIES Total score and subscales, all correlations were significant at the *p* < .001 level. Overall, as seen in Table 4, all correlations were positive and medium to large using standard cut-offs (i.e., .290–.636), indicating that as each of the participant endorsements of stress reactions (i.e., Intrusion, Avoidance, and Arousal) increased, so too did the correlated mental health outcomes (i.e., Conduct, Negative Affect, and Cognitive/Attention). The strongest correlations were between the CRIES Arousal subscale and the BIMAS Negative Affect and Cognitive/Attention subscales.

**Table 4.** Correlations between the BIMAS Behavioral Concern Scales and CRIES Subscale Scores.

CRIES subscales	BIMAS behavioral concern scales		
	Conduct	Negative affect	Cognitive/attention
Intrusion	0.324	0.437	0.318
Avoidance	0.290	0.376	0.265
Arousal	0.482	0.636	0.576

#### Multivariate analysis

To determine which stress reactions accounted for the greatest variance in mental health outcomes, hierarchical multivariate regressions were completed. Age and gender were entered in Block 1, and the three CRIES subscale scores in Block 2, with each of the Behavioral Concern Scales as the subsequent dependent variables. In these models, the three CRIES subscales significantly predicted each of the three BIMAS Behavioral Concern Scales. In predicting Conduct, gender (male, β = –.039, *t*(2,203) = –2.07, *p* < .05) negatively and Arousal (β = .473, *t*(2,203) = 18.60, *p* < .001) positively predicted Conduct scores (*R*^2^ = .25, *F*(4, 2,203) = 145.18), such that being male and higher Arousal scores accounted for 25% of the variance in Conduct problems in this sample. In predicting Negative Affect, including mood and anxiety, the combination of gender (female, β = .172, *t*(2,203) = 11.28, *p* < .001), Intrusion (β = .048, *t*(2,203) = 2.19, *p* < .05), and Arousal (β = .597, *t*(2,203) = 29.38, *p* < .001) positively predicted Negative Affect scores (*R*^2^ = .52, *F*(4, 2,203) = 477.00). In other words, females had higher Negative Affect scores (as compared to males), and higher Arousal and Intrusion scores accounted for 52% of the variance in Negative Affect. Finally, in predicting Cognitive/Attention functioning, age (15–18 years, β = .072, *t*(2,203) = 4.23, *p* < .001) and Arousal (β = .653, *t*(2,203) = 28.643, *p* < .001) positively predicted Cognitive/Attention scores (*R*^2^ = .40, *F*(4, 2,203) = 287.33), such that older youths’ (15–18 years of age) had higher Cognitive/Attention scores (as compared to 12–14 year-olds), and higher Arousal scores accounted for 40% of the variance in Cognitive/Attention functioning (i.e., lowered).

## Discussion

Best estimates from North American statistics indicate that children and youth represent less than 10% of the SARS-CoV-2 symptomatic infections (Centers for Disease Control and Prevention (CDC), 2020). There has been significant concern, however, about the potential negative impact on children and adolescents of the many societal effects of the pandemic, including school closures, social distancing, and threats to the health of loved ones. This has led to “best practice” guidelines (e.g., National Association of School Psychologists (NASP), 2020; Science & Bitnum, 2020) for schools, families, and students as they planned their return-to-school approaches. As helpful as these directives are to schools and students, longitudinal, real-time studies are necessary to add data to evidence-informed decision-making during COVID-19 (Fegert & Schulze, 2020).

To this end, this paper represents preliminary results of Wave 1 data collection from an ongoing, 1-year longitudinal study; participants will also be asked to participate in three other waves of data collection during the 2020–2021 school year to longitudinally explore the associations reported in this paper. In these baseline data (collected in fall 2020, at the same time as school re-opening in Alberta), we found that adherence to COVID-19 public health measures was good overall. For example, almost two-thirds of participants reported that they engaged in physical distancing all or most of the time. However, there were some differences by gender and age. In particular, older adolescents were more likely to say that they had socialized with someone outside of their bubble, and were less likely to adhere to physical distancing guidelines, as compared to younger adolescents. This may reflect the increased salience of peer relationships to adolescents with age (Steinberg, 2020). Conversely, female participants were less likely than male participants to state that they maintained distance with people outside their household, which again may represent the stronger socialized need to form and maintain interpersonal relationships for cisgender females in Western culture (Schwartz-Mette et al., 2020). Together, these findings suggest a need for age- and gender-targeted public health messaging around COVID-19 public health orders in Canada.

A large concern in this pandemic has been the anticipated “second wave” of mental health issues (e.g., due to the isolation youth have faced as a result of school closures). However, our data suggest that overall, youth in our sample were doing quite well 6 months into the pandemic, and that differences in mental health may reflect pre-existing disparities. Specifically, and guided by cut-off scores from previous studies (e.g., Perrin et al., 2005), the level of posttraumatic stress reactions (via the CRIES-13 measure) reported by our sample was far below the critical cut-off. Looking at the specific components of participant’s experience of the traumatic event (i.e., COVID-19), we found the highest scores for Arousal (though again, this score was far below the critical cut-off) as compared to Avoidance and Intrusion. This aligns with research showing that traumatic events like COVID-19 are processed in a negative way, arousing emotions that lead to a sense of serious and current threat (e.g., Ehlers & Clark, 2000). We also found that female participants and older participants had significantly higher stress reaction scores overall, and on the three subscales (Intrusion, Avoidance, Arousal). Research on stressful life events has found that females who are exposed to stressors that involve the well-being of significant others (i.e., family, friendships) have amplified responses to those stressors (Lavoie et al., 2019). Our results certainly indicate that COVID-19 is perceived as a threat to both health and social relationships by some youth, and that females in particular may be more attuned to these dynamics as represented by their higher stress subscale scores.

In terms of mental health, scores for all three Behavioral Concern Scales fell in the low-risk level for the majority of students, suggesting that despite the pandemic, youth are functioning quite well overall. As with stress reactions, however, females and older youth report significantly higher scores on the measures of negative affect (e.g., being sad or withdrawn) and cognitive/attention (e.g., had trouble remembering things). Older youth also reported fewer conduct behaviors (e.g., feeling angry) than younger youth, and although female youth reported higher conduct scores than male youth, being male was a significant predictor of higher Conduct scores (Rosenfeld & Mouzon, 2013). The gender difference in conduct scores was also maintained in multivariate models.

Associations between stress and mental health in our sample were, as expected, positive, and in the medium to large range. The largest correlations (>*r* = .50) were between CRIES Arousal and two Behavioral Concern Scales, Negative Affect and Cognitive/Attention. Recent studies exploring the association between COVID-related stress and mental health have also found similar moderate correlations (i.e., *r/span* ≥ .49–.54), suggesting that hypervigilance and increased nervousness are robustly correlated with negative changes in cognition and feelings (Taylor et al., 2020). In multivariate models, arousal significantly predicted conduct, negative affect (i.e., mood/anxiety), and cognitive/attention functioning scores, controlling for age and gender. In addition, intrusion significantly predicted negative affect scores. These models also accounted for a substantial amount of variance in mental health outcomes (between 25% and 50%), suggesting that stress is an important predictor of mental health for this sample during their return to school in fall.

### Relevance to the Practice of School Psychology

Mental health professionals are invested in how children and youth return to school following COVID-19. The absence of data on both the strengths (resilience) and needs (mental health problems) of students in the context of the pandemic makes the provision of ethical and accurate programming incredibly difficult for all learners, including those with diverse learning needs. Based on the findings of this study, we present three key take-aways for school mental health practitioners. First, while students overall appear to be doing as well as would be expected developmentally, there are sub-groups who require continued support. From our data, two important sub-groups are cisgender female youth and older youth. From an equity perspective, practitioners should consider which youth in their context may be most vulnerable to the pandemic and associated impacts (e.g., youth whose parents are frontline workers; racialized youth), and consider offering targeted support to these youth. Perhaps the best way to consider the student-in-context is described by Prime and colleagues (Prime et al., 2020), wherein the adolescent is situated within processes of both risk and resilience. Most concerning, however, is that there are cumulative risks to which the adolescent is exposed—loss of social contact, irregular curriculum delivery, threat of illness—and knowing what resilience factors might be activated similar to other global disasters (Masten & Narayan, 2012) is still to be determined. Second, schools should consider using age- and gender-targeted messaging when encouraging students to follow public health orders. For example, with older adolescents, school mental health practitioners could focus messaging on how most youth their age are following guidelines (e.g., a social norms approach). Finally, as these are cross-sectional data, and as such offer only a snapshot of student well-being, it is important to continue assessing the impact of the pandemic on student stress and mental health. For this purpose, school mental health practitioners could consider the adoption of universal screening measures (Allen et al., 2019; Romer et al., 2019). We are currently collecting the second wave of these data, and thus will also be able to explore mental health over time, as well as longitudinal associations between school engagement (e.g., online vs. in-person) and well-being.

### Limitations

While this study provides a thorough overview of youth stress and mental health during school return in fall 2021 in one province, there are several limitations. First, and as mentioned previously, these data are cross-sectional, and thus associations should not be interpreted as causal. We are currently collecting additional waves of data throughout the 2020–2021 school year and will be able to present longitudinal associations in the near future. Second, while we can speak to youth stress and mental health in one province at an aggregate level, and in terms of certain subgroups, we recognize that some youth may need additional support following the pandemic. For example, our sample only captures adolescents who lived in large, metropolitan western Canadian cities, and associations may be very different for rural youth where supports are more limited. Third, we recognize that we used scales (e.g., CRIES) that were not constructed for measuring stress reactions during a pandemic, nor was our measure of behavioral and mental health (i.e., BIMAS-2) designed to estimate responses from youth during a time of so much volatility and magnitude of change. Their utility here hopefully serves as best approximations of students’ self-reports during very extraordinary personal and global times. Finally, all data were self-report, and were not corroborated by parent/guardian or teacher reports.

## Conclusion

School mental health practitioners need information on how youth are faring in the midst of the COVID-19 pandemic. The findings from the first wave of a longitudinal study focused on youth well-being suggest that on average youth are doing about as expected, but that certain subgroups are in need of additional support. We will use future longitudinal data collected as part of this study to further explore and extend the present findings.
